# Application of ecosystem-specific reference databases for increased taxonomic resolution in soil microbial profiling

**DOI:** 10.3389/fmicb.2022.942396

**Published:** 2022-11-03

**Authors:** Christina Karmisholt Overgaard, Ke Tao, Sha Zhang, Bent Tolstrup Christensen, Zuzana Blahovska, Simona Radutoiu, Simon Kelly, Morten Kam Dahl Dueholm

**Affiliations:** ^1^Department of Chemistry and Bioscience, Center for Microbial Communities, Aalborg University, Aalborg, Denmark; ^2^Department of Molecular Biology and Genetics, Aarhus University, Aarhus, Denmark; ^3^Department of Agroecology, Aarhus University, AU-Foulum, Tjele, Denmark

**Keywords:** soil, rhizosphere, microbiota, host preference, 16S rRNA (16S rDNA)

## Abstract

Intensive agriculture systems have paved the way for a growing human population. However, the abundant use of mineral fertilizers and pesticides may negatively impact nutrient cycles and biodiversity. One potential alternative is to harness beneficial relationships between plants and plant-associated rhizobacteria to increase nutrient-use efficiency and provide pathogen resistance. Plant-associated microbiota profiling can be achieved using high-throughput 16S rRNA gene amplicon sequencing. However, interrogation of these data is limited by confident taxonomic classifications at high taxonomic resolution (genus- or species level) with the commonly applied universal reference databases. High-throughput full-length 16S rRNA gene sequencing combined with automated taxonomy assignment (AutoTax) can be used to create amplicon sequence variant resolved ecosystems-specific reference databases that are superior to the traditional universal reference databases. This approach was used here to create a custom reference database for bacteria and archaea based on 987,353 full-length 16S rRNA genes from Askov and Cologne soils. We evaluated the performance of the database using short-read amplicon data and found that it resulted in the increased genus- and species-level classification compared to commonly use universal reference databases. The custom database was utilized to evaluate the ecosystem-specific primer bias and taxonomic resolution of amplicon primers targeting the V5–V7 region of the 16S rRNA gene commonly used within the plant microbiome field. Finally, we demonstrate the benefits of custom ecosystem-specific databases through the analysis of V5–V7 amplicon data to identify new plant-associated microbes for two legumes and two cereal species.

## Introduction

A growing world population necessitates continued improvements in agricultural output to ensure food security. Current agricultural practices may involve excessive applications of mineral fertilizers and pesticides, which need to be reduced and supplemented with more sustainable solutions (Tilman et al., 2011). One proposed solution is to harness the capabilities of natural soil microbes (Mendes et al., 2013; Toju et al., 2018; Trivedi et al., 2020). Developing the potential benefits of microbes requires a deeper understanding of the interactions occurring between plants and microbes at a community level within the complex soil environment.

Initial investigations of plant microbiomes largely relied on the cultivation of microbes, limiting these investigations to the easily culturable fraction of the microbial communities (Chelius and Triplett, 2001). However, advances in DNA sequencing technologies and bioinformatic tools have allowed large-scale amplicon-based microbiome studies to become commonplace, allowing for more comprehensive detection of the microbes present. In terms of plant–microbe interactions, studies have utilized amplicon sequencing to investigate the microbial diversity of soils retrieved from various ecosystems such as tallgrass prairie, tundra, tropical rainforest, and agroecosystems (Tripathi et al., 2012; Fierer et al., 2013; Gittel et al., 2014; Armalyte et al., 2019). Through profiling of root-associated communities, key insights have been gained into the factors that govern the assembly of the root microbiome including soil type, root exudates, plant genotype, plant developmental state, and season (Jacobs et al., 2011; Zgadzaj et al., 2016; Stringlis et al., 2018; Finkel et al., 2019; Huang et al., 2019; Thiergart et al., 2019; Voges et al., 2019). The 16S rRNA V5–V7 primer pair (Nocker et al., 2010; Bonder et al., 2012; Buchholz et al., 2019) is often used for bacterial profiling in plant studies because amplicons originating from plant chloroplast and mitochondria can be minimized *via* size selection and primer specificity. Without the exclusion of host-derived amplicons, the fraction of microbial amplicons is greatly diminished, resulting in a skewed picture of the microbial community (Beckers et al., 2016).

One of the major limiting factors in the analysis of the data generated through amplicon-based microbiome studies is taxonomic assignment. To understand the biological roles of soil microbes, we need to be able to identify them correctly and comprehensively. However, most amplicons are only classified at the family level or above with commonly applied reference databases (Dueholm et al., 2020). This is problematic as many important physiological traits only are conserved at the genus level or below (Martiny et al., 2015).

To overcome this, ecosystem-specific databases can be created using a combination of high-throughput full-length 16S rRNA gene sequencing and automated taxonomy assignment (AutoTax) (Dueholm et al., 2020). Such databases improve the taxonomic classification of both short- and long-read amplicon sequence variants (ASVs), providing increased taxonomic classification at the genus- and species level (Dueholm et al., 2020, 2022).

Here, we demonstrate how ecosystem-specific reference databases can be used for exploratory studies to increase the taxonomic resolution in soil microbial profiling studies, leading to new insight. For this, we sequenced 987,353 full-length 16S rRNA genes from two soils commonly used for plant microbiome research: Askov soil, which has been used for plant and soil studies for more than 125 years (Christensen et al., 2019), and Cologne soil, which has been used in numerous plant–microbe interaction studies (Bulgarelli et al., 2012; Zgadzaj et al., 2016; Thiergart et al., 2019, 2020; Wippel et al., 2021). The full-length 16S rRNA genes were processed with AutoTax to create a reference database composed of 18,042 ASV-resolved full-length 16S rRNA genes (FL-ASVs) with a complete seven-rank taxonomy (domain to species) for all reference sequences. We use the ecosystem-specific database to (i) improve the classification of V5–V7 amplicons from Askov soil, rhizosphere, and endosphere at genus- and species level, (ii) uncover ecosystem-specific primer bias associated with the V5–V7 primer set, and (iii) investigate the host preference of bacterial taxa in associations with two legumes (*Lotus japonicus* and *Medicago truncatula*) and two cereal (*Hordeum vulgare* and *Zea mays*) species.

## Materials and methods

### Soil and plant materials

The Askov soil was obtained from the Askov Experimental Station situated in Southern Denmark (GPS coordinates: 55.466 N, 9.117 E). The soil was sampled in 2018 from 0- to 20-cm soil depth in three replicate plots (plot no. 421, 443, and 474) in the B4 field. This field is part of the Askov Long-Term Experiment established in 1894, and the plots sampled for this study have been kept without manure and fertilizer application since then. The soil is a light sandy loam with 11% clay, 13% silt, and 76% sand and classifies as Alfisol (Typic Hapludalf, USDA Soil Taxonomy). The soil grows a four-course rotation of winter wheat, silage maize, spring barley, and a grass-clover mixture used for cutting. The addition of lime every 4–5 years keeps soil pH in the range of 5.5–6.5 (Christensen et al., 2019). Cologne soil was obtained from the Max Planck Institute for Plant Breeding Research in Cologne, Germany (GPS coordinates: 50.958 N, 6.856 E) and has not been in agricultural use for over 15 years (Bai et al., 2015; Harbort et al., 2020). The Cologne soil used was collected in Spring 2017 from a depth of 15–30 cm. The soil was dried at room temperature for 1 week followed by storage in the dark at 4°C. Characteristics of Cologne soil have previously been reported (Bulgarelli et al., 2012).

Seeds of *Lotus japonicu*s Gifu (*Lj*) (Handberg and Stougaard, 1992) and *Medicago truncatula* A17 (*Mt*) (Young et al., 2011) were available from our stocks at Aarhus University. *Hordeum vulgare* cv. Golden promise (*Hv*) and *Zea maise* cv. W22 (*Zm*) were received from the Crop Science Centre, University of Cambridge.

### Plant setup and harvesting

Seeds were surface sterilized in a 1:20 bleach solution for 5–15 min, washed five times in sterile H_2_O, and germinated on wet filter paper for 2–5 days. Seedlings were aseptically transferred to sterile pots (12 cm high, 13 cm diameter) that had been filled with ~350 g Askov soil (*Lj* and *Mt* 10 plants/pot; Hv and Zm 4 plants/pot). Three pots were set up for each plant species. Plants were grown in a growth chamber under controlled conditions: 16/8 h day/night, 75% humidity, 22°C (day), and 18°C (night). Watering throughout the experiment was with sterile H_2_O only.

After 3 weeks of growth, at which point mature nitrogen-fixing nodules had formed on the legumes, plants were removed from pots, and roots separated from shoots. Root material was vortexed for 30 s in 50 ml Falcon tubes containing 35 ml sterile H_2_O, and roots were transferred to a new Falcon tube. The initial Falcon tube was centrifuged (4,000 × *g*, 15 min), and most supernatants were removed. The soil pellet was resuspended in the remaining liquid (ca. 2 ml) using a cut P1000 tip; 300 μl of this suspension was transferred to a Lysis matrix E tube (MP Biomedicals) forming the rhizosphere fraction. The root material was washed three times in sterile H_2_O for 30 s, once in detergent (1× TE + 0.1% Triton X-100) for 2 min, once in 80% ethanol for 30 s, once in 3% bleach for 30 s and finally five times in sterile H_2_O. The surface sterilized root material was then transferred to Lysis matrix E tubes forming the endosphere fraction. For *Lj* and *Mt*, nodules were removed from sterilized root material and collected in separate Lysis matrix E tubes forming the nodule fraction. Details about all samples can be found in Supplementary Data S1.

### General molecular methods and DNA extraction

Concentrations of DNA were measured using a Qubit 3.0 fluorometer (Thermo Fisher Scientific), and quality was determined using Agilent 2200 Tapestation (Agilent Technologies). AMPure XP beads were used for DNA cleanup in accordance with manufacturer's protocol except for the washing step, where 80% ethanol was used. All commercial kits were used according to manufacturer's protocol unless otherwise stated.

DNA was extracted using the FastDNA Spin Kit for Soil (MP Bio) with the homogenization performed using a Precellys tissue lyser (Bertin Instruments) for 2 × 30 s at 6,000 rpm or using the RNeasy PowerSoil Total RNA Kit with the PowerSoil DNA Elution Kit (Qiagen). Quality was determined by tapestation using a genomic DNA ScreenTape. Concentrations were measured using Qubit™ dsDNA HS Assay Kit.

### Full-length 16S rRNA gene library preparation and sequencing

Full-length 16S rRNA gene sequencing was essentially carried out as described in Dueholm et al. (2022) with the addition of an extra primer pair targeting archaeal 16S rRNA genes. Minor deviations from the original protocol were introduced to accommodate the specific sample type (soil) and improve the robustness of the protocol. A detailed description of the method is provided below. Oligonucleotides used can be found in Supplementary Table S1. Sequencing libraries were prepared from the following sample types: Askov and Cologne bulk soil and rhizosphere of *Lj, Mt, Hv*, and *Zm* grown in Askov soil for 3 weeks and *Lj* rhizosphere grown in Cologne soil for 3 weeks (Supplementary Data S1).

#### Adaptor annealing by PCR

Adaptors containing sample barcodes, unique molecular identifiers (UMI), and defined primer binding sites were added to each end of the bacterial 16S rRNA genes by PCR. The reaction contained 10 μl of 10× PCR Buffer (Qiagen), 2 μl of 10 mM dNTP (Qiagen), 5 μl of 10 μM f16S_pcr1_fw, 5 μl of 10 μM f16S_pcr1_rv, 4 μl (bacteria) or 8 μl (archaea) of 25 mM MgCl_2_, 0 μl (bacteria) or 20 μl (archaea) of 5× Q-solution (Qiagen), 0.5 μl of 5 U/μl Taq polymerase (Qiagen), 100 ng of template DNA, and nuclease-free water to 100 μl. The reaction was incubated with an initial denaturation at 94°C for 3 min followed by 2 cycles of denaturation at 94°C for 30 s, annealing at 56°C (bacteria) or 54°C (archaea) for 30 s, and extension at 72°C for 3 min, and then a final extension at 72°C for 5 min. The sample was purified using 0.6× AMPure XP beads and eluted in 21-μl nuclease-free water.

The following versions of the f16S_pcr1_fw forward primers were used: Askov rhizosphere (fw1), Askov bulk soil (fw2), Cologne rhizosphere (fw3), and Cologne bulk soil (fw4). The f16S_pcr1_rv1 reverse primer was used for all samples.

#### Primary library amplification

The adaptor-annealed 16S rRNA gene amplicons were amplified using PCR to obtain enough product for quantification and sequencing. The reaction contained 19 μl of adaptor annealed sample, 20 μl of 5× Phusion HF (New England Biolabs), 2 μl of 10 mM dNTP, 5 μl of 10 μM f16S_pcr2_fw, 5 μl of 10 μM f16S_pcr2_rv, 4 μl of 25 mM MgCl_2_, 44 μl nuclease-free water, and 1 μl 2U/μl Phusion HF DNA polymerase (NEB). The reaction was incubated with an initial denaturation at 98°C for 30 s followed by 15 (bacteria) or 20 (archaea) cycles of denaturation at 98°C for 10 s, annealing at 62°C for 30 s, and extension at 72°C for 1 min, followed by a final extension at 72°C for 5 min. DNA was eluted in 11 μl nuclease-free water. Quality was determined using a D5000 ScreenTape and concentrations were measured using Qubit™ dsDNA HS Assay Kit. Libraries for the different sample barcodes were pooled with an equal amount of DNA from each sample.

#### Clonal library amplification

Adaptor annealed amplicon libraries were diluted to ~200,000 molecules/μl and amplified by PCR to obtain clonal copies of each uniquely tagged amplicon molecule. The PCR reaction contained 63.5 μl nuclease-free water, 10 μl of 10× PCR buffer (Qiagen), 2 μl of 10 mM dNTP, 5 μl of 10 μM f16S_pcr2_fw, 5 μl of 10 μM f16S_pcr2_rv, 4 μl of 25 mM MgCl_2_, 0.5 μl of 5 U/μl Taq polymerase (Qiagen) and 10 μl diluted adaptor annealed amplicon product. The reaction was initiated by denaturation at 94°C for 3 min, followed by 20 cycles of denaturation at 94°C for 30 s, annealing at 62°C for 30 s and extension at 72°C for 2 min, followed by a final extension at 72°C for 5 min. The PCR product was purified using 0.6× AMPure XP beads with elution into 21 μl nuclease-free water. The product quality and concentration were analyzed on a D5000 screen tape and with the Qubit dsDNA HS Assay Kit, respectively.

#### Read-tag library preparation

A Nextera library preparation kit (Illumina) was used to prepare a paired-end read-tag sequencing library from the clonal library using a customized protocol. A tagmentation reaction was prepared with 100 ng of the clonal library in 22.5 μl nuclease-free water, 25 μl tagment DNA buffer (Illumina), and 2.5 μl tagment DNA enzyme (Illumina). The reaction was incubated at 55°C for 5 min. The product was immediately diluted to 100 μl and purified using 0.6× AMPure XP beads with elution into 42 μl nuclease-free water.

The tagmentation products were PCR amplified using two separate PCRs (A and B). PCR A selectively amplified fragments containing the 5′ termini of the amplicons and PCR B selectively amplified fragments containing the 3′ termini. The reactions contained 20 μl purified tagmentation product, 5 μl N504 Nextera adaptor (Illumina), 5 μl of 10 μM f16S_readtag_fw (PCR A) or f16S_readtag_rv (PCR B), 5 μl PCR primer cocktail (Illumina), 10 μl of 5× Phusion HF buffer (NEB), 1 μl of 10 mM dNTP, 3.5 μl nuclease-free water, and 0.5 μl of 2U/μl Phusion HF DNA polymerase (NEB). The following PCR program was used: Initial elongation at 72°C for 3 min, initial denaturation at 98°C for 30 s, and 10 cycles of denaturation at 98°C for 10 s, annealing at 60°C for 30 s and elongation at 72°C for 3 min, followed by a final extension at 72°C for 5 min. The raw read-tag libraries were purified using 1.0× AMPure XP beads with elution into 21-μl nuclease-free water.

To ensure even sequencing coverage across the length of the 16S rRNA gene amplicons, the size distribution of the read-tag libraries was optimized (Karst et al., 2018). The libraries were size fractionated on an E-Gel CloneWell gel (Thermo Fisher Scientific). A total of 500 ng GeneRuler 1 kb DNA ladder (Thermo Fisher Scientific) was used as a length reference. The gel was run until the 500 bp marker was 1 mm from the elution well, after which 20-μl elution aliquots were sampled and replaced by nuclease-free water every 15 s, up to a total of 32 aliquots. Every two aliquots were pooled, yielding 16 pooled aliquots per sample. These were then analyzed on an Agilent 2200 Tapestation using the High Sensitivity D1000 ScreenTape. Fractions with a mean fragment length of 500–1,250 bp were used for the pooling. The effective sequencing concentration for fractions from 500 to 950 bp was determined based on the tapestation data and the empirical formula C_seq_ = Peak molarity [pmol/l] ^*^ (−0.0124^*^(peak size [bp] – 215 bp) + 10.332) (Karst et al., 2018). These fractions were pooled with equal effective sequencing concentration (C_seq_). For fractions between 950 and 1,250 bp, the entire aliquot was used for pooling (40–50 μl). The pooled aliquots were then purified using 1.0× AMPure XP beads with elution into 11 μl nuclease-free water. The quality and concentration of the coverage-optimized read-tag libraries were analyzed on D1000 screen tapes and with the Qubit dsDNA HS Assay Kit, respectively.

#### Linked-tag library preparation

Clonal libraries were end-repaired in a reaction containing 20 ng clonal library, 2.5 μl of 10 × NEBNext End Repair Reaction Buffer (New England Biolabs), 1.25 μl NEBNext End Repair Enzyme Mix (New England Biolabs), and nuclease-free water to 25 μl. The reaction was incubated at 20°C for 30 min. The end-repair reaction was purified using 1.0× AMPure XP beads and eluted into 10 μl nuclease-free water.

The end-repaired sample was circularized in an intramolecular blunt end ligation reaction containing 150 μl nuclease-free water, 20 μl of 50% (w/w) PEG 4000 solution (Thermo Fisher Scientific), 20 μl of 10× T4 DNA ligase buffer (NEB), 8 μl of T4 DNA ligase (NEB), and 2 μl of the end-repaired clonal library. The reaction was incubated at 16°C for 60 min. The circularized products were purified using 1.0× AMPure XP beads and eluted in 10 μl nuclease-free water.

The junction sequence, which contains both UMI tags, was amplified by PCR in a reaction containing 8 μl of circularized clonal library, 5 μl of 10× PCR buffer (Qiagen), 1 μl of 10 mM dNTP mix, 2.5 μl of 10 μM f16S_linktag_fw, 2.5 μl of f16S_linktag_rv, 2 μl (bacteria) or 4 μl (archaea) 25 mM MgCl2, 0 μl (bacteria) or 10 μl (archaea) 5 × Q-solution (Qiagen), 0.25 μl 5 u/μl Taq polymerase (Qiagen), and nuclease-free water to 50 μl. The PCR reaction was initiated by denaturation at 94°C for 3 min, followed by 20 cycles of denaturation at 94°C for 20 s, annealing at 56°C (bacteria) or 54°C (archaea) for 20 s, and extension at 72°C for 20 s, followed by a final extension at 72°C for 3 min. The PCR product was purified using 1.0 × AMPure XP beads and elution into 12 μl nuclease-free water. The quality and concentration of the linked-tag libraries were analyzed on D1000 ScreenTape and with the Qubit dsDNA HS Assay Kit, respectively.

#### Library pooling

The coverage normalized read-tag libraries A and B were diluted to 0.9 ng/μl. The linked-tag library was diluted to 0.2 ng/μl. The libraries were pooled by combining 4.6 μl read-tag library A, 4.6 μl read-tag library B, and 0.8 μl linked-tag library.

#### Sequencing

The libraries were paired-end (1 × 240 bp and 1 × 25 bp) sequenced on a HiSeq 2500 instrument (Illumina) using on-board clustering and rapid run mode with a HiSeq PE Rapid Cluster Kit v2 (Illumina) and HiSeq Rapid SBS Kit v2, 200 cycles (Illumina). The SBS reagents were supplemented with 9.5 ml Incorporation Master Mix, 9.5 ml Cleavage Reagent Mix, and 7 ml Universal Scan Mix to enable sequencing of 265 cycles. The HiSeq was running HiSeq Control Software v.2.2.68 (Illumina) and Real Time analysis v.1.18.66.3 (Illumina). The libraries were prepared and loaded on the HiSeq using the standard procedures (Illumina: manual # 15035786 v01; manual # 15050107 v.02; manual # 15061846 v.01) with the following changes. A volume of 10 μl library pool was denatured by adding 10 μl of 0.1 N NaOH solution, mixing well by pipetting, and incubating for 5 min at 25°C. The denatured library pool was diluted by adding 980 μl of cold Hybridization Buffer (Illumina); 400 μl of the denatured and diluted library pool was mixed with 20 μl of denatured and diluted 10 pM PhiX control v3 library (Illumina) and stored on ice until loading. Custom read2 primer mix was prepared by mixing 25 μl of 100 μM f16S_read2_fw and 25 μl of 100 μM f16S_read2_rv in a conical tube (15 ml) and diluting with 4,950 μl Hybridization Buffer (final concentration 0.5 μM). When the paired-end reagent rack was loaded on the HiSeq, the Illumina primer mix in position nr. 16 was replaced with the custom read2 primer mix prepared above. When setting up the HiSeq run in the control software, the standard procedure was followed except for the following steps: For the “Recipe Screen”, the following options were chosen: Index type options = No Index; Read 1 cycles = 240; Read 2 cycles = 25. After sequencing, bcl2fastq v.2.17.1.14 (Illumina) was used to generate fastq files from bcl files using standard settings (manual # 15038058 RevB).

### Assembly of full-length 16S rRNA genes and generation of the AsCoM database

Raw sequence reads were binned, based on the UMIs, and *de novo* assembled into the synthetic long-read rRNA gene sequences as previously described (Dueholm et al., 2020). The assembled 16S rRNA gene sequences were oriented based on the SILVA 138 SSURef Nr99 database using the usearch v.11.0.667-orient command and trimmed between the 27f and 1391r primer binding sites (bacteria) or SSU1ArF and SSU1000ArR primer binding sites (archaea) using the trimming function in CLC genomics workbench v.20.0. Sequences without both primer binding sites were discarded. The trimmed full-length 16S rRNA genes were processed with AutoTax v.1.5.2 (Dueholm et al., 2020) to create the FL-ASV resolved AsCoM reference database and taxonomy.

### Construction of phylogenetic trees

The FL-ASVs aligned to the SILVA_138.1_SSURef_NR99 ARB database (AutoTax: temp/FL-ASVs_SILVA_aln.fa) were loaded into ARB, and the AutoTax generated taxonomy was added to the sequences (AutoTax: output/tax_complete.csv) after being concatenated in excel. All bacterial or archaeal sequences were hereafter exported from ARB as a fasta alignment using the positional variability by parsimony filter ssuref:bacteria or ssuref:archaea. Phylogenetic trees were constructed using FastTree v.2.1.11 (Price et al., 2010) with the -gtr and -nt options.

### 16S rRNA V5–V7 amplicon sequencing

A two-step PCR protocol was used to amplify the 16S rRNA V5-V7 region and index samples using the 799F (5′-AACMGGATTAGATACCCKG-3′) and 1192R (5′-ACGTCATCCCCACCTTCC-3′) primers as described previously (Wippel et al., 2021). Paired-end 350 bp sequencing was performed by IMGM Laboratories GmbH (www.imgm.com) on the Illumina MiSeq platform.

16S rRNA gene V5–V7 forward and reverse reads were processed using usearch v.11.0.667 (Edgar, 2010). Forward and reverse reads were merged using the usearch -fastq_mergepairs command, filtered to remove phiX sequences using usearch -filter_phix, and quality filtered using usearch -fastq_filter with -fastq_maxee 1.0. Dereplication was performed using -fastx_uniques with -sizeout, and amplicon sequence variants (ASVs) were resolved using the usearch -unoise3 command. An ASV table was created by mapping the quality filtered reads to the ASVs using the usearch -otutab command with the -zotus and -strand plus options. Taxonomy was assigned to ASVs using the specified reference databases and the usearch -sintax command with -strand both and -sintax_cutoff 0.8 options. Mapping of ASVs to reference databases was done with the -usearch_global command and the -id 0, -maxaccepts 0, -maxrejects 0, -top_hit_only, and -strand plus options unless otherwise stated.

### Evaluation of ecosystem-specific primer bias

The phylogenetic signal of V5–V7 amplicons was evaluated based on *in silico* ASVs extracted from the aligned FL-ASVs in ARB. FL-ASVs were trimmed between the base pair positions after the end of the forward primer and before the start of the reverse primer. A fasta file containing the redundant set of *in silico* ASVs were classified using the AsCoM database using usearch -sintax with -strand plus and -sintax_cutoff 0.8. The classifications were compared to those of the parental FL-ASVs in the AsCoM database.

The ecosystem-specific primer bias associated with the V5–V7 primer bias was evaluated using the bacterial FL-ASVs in the AsCoM database and the analyze_primers.py script from Primer Prospector v. 1.0.1 (Walters et al., 2011). The specificity of primer sets was defined based on the overall weighted scores (OWSs) for the primer with the highest score as follows: perfect hit (OWS is 0), partial hit (OWS is >0 and ≤ 1), and poor hit (OWS is >1). The scores are based on the following criteria: The last five bases are considered the 3', non 3' mismatch penalty = 0.40 per mismatch, 3' mismatch penalty = 1.00 per mismatch, last base mismatch penalty = 3.00, non 3' gap penalty = 1.00 per gap, and 3' gap penalty = 3.00 per gap.

## Results

### Establishment of an ecosystem-specific 16S rRNA gene reference database

To create an ecosystem-specific reference database applicable for both Askov and Cologne soils (AsCoM), we applied synthetic long-read sequencing to obtain near full-length bacterial and archaeal 16S rRNA genes using DNA from bulk soil and rhizosphere samples of plants grown in the two soils. Approximately the same number of sequences were obtained for each soil, resulting in a total of 987,353 full-length 16S rRNA gene sequences after primer and quality filtering. These sequences were subsequently processed using AutoTax (Dueholm et al., 2020) to resolve FL-ASVs, and create a comprehensive taxonomy for all sequences. AutoTax incorporates *de novo* placeholder names (AsCoM_x_y) for the many uncultured environmental taxa, which are not yet taxonomically classified at all seven ranks (Dueholm et al., 2020). The result of this is a taxonomy that can be interrogated at even the lowest taxonomic ranks (species level) for all reference sequences. This is not possible when conventional reference databases are used to assign taxonomy due to missing taxonomic assignments, particularly in the lower ranks. The final AsCoM database contained 18,042 unique FL-ASVs, each with a complete seven-rank taxonomy assigned.

### AsCoM reveals great taxonomic novelty within the soil community

**Figure 1:**
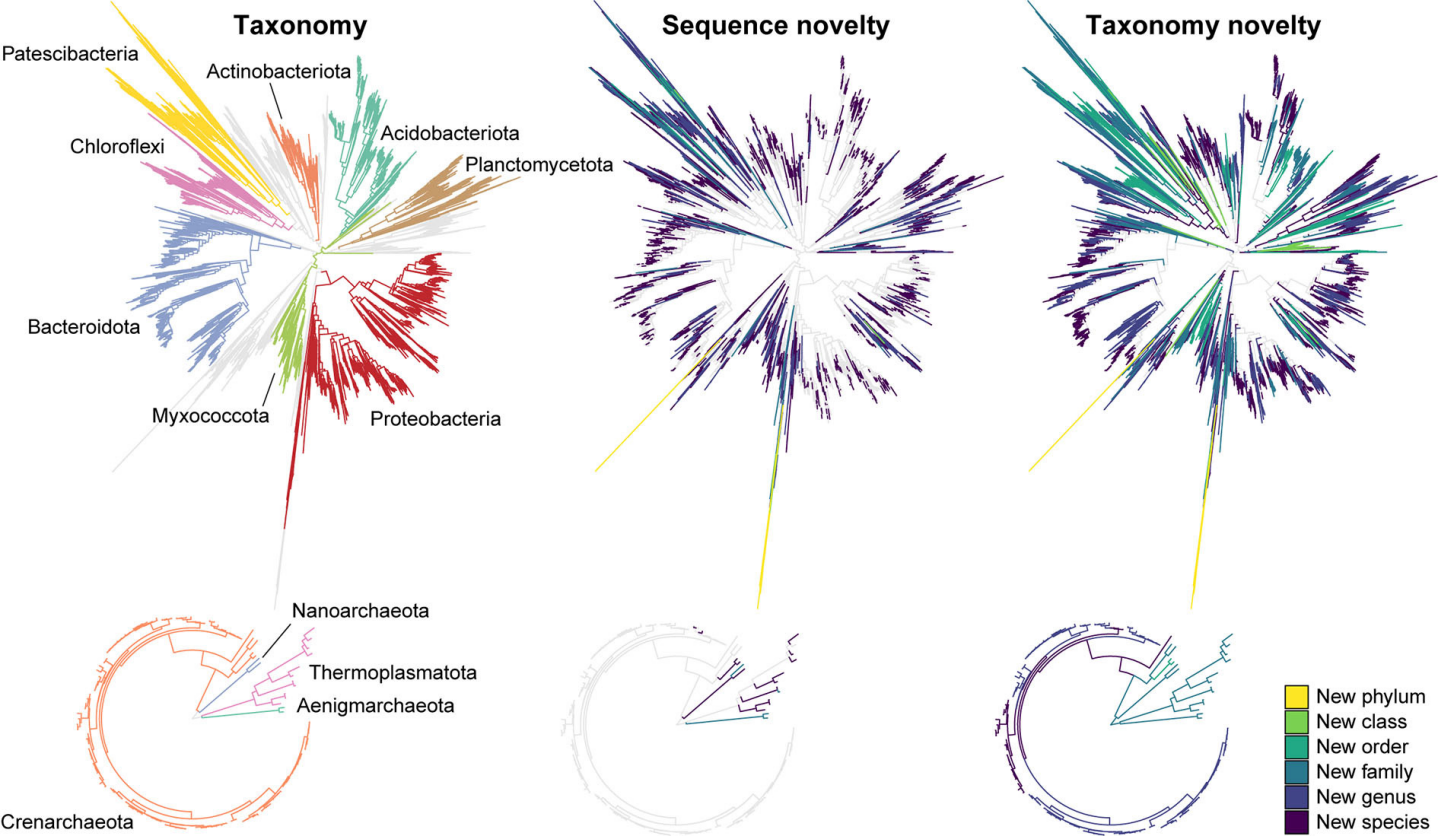
Novel sequences and *de novo* taxa observed in the AsCoM reference database. Phylogenetic trees are based on the FL-ASVs in the AsCoM database. The eight phyla with the most FL-ASVs are highlighted. Sequence novelty was determined by the percent identity between each FL-ASV and their closest relative in the SILVA_138.1_SSURef_Nr99 database and taxonomic thresholds proposed by Yarza et al. (2014) shown in Table 1. Taxonomy novelty was defined based on the assignment of *de novo* taxa by AutoTax (Dueholm et al., 2020).

The sequence-based novelty of the AsCoM reference sequences was evaluated by mapping the FL-ASVs to the SILVA_138.1_SSURef_NR99 database (Quast et al., 2013) and determining the percentage of FL-ASVs that have reference sequences within the identity thresholds for each taxonomic rank suggested by Yarza et al. (2014) (Figure 1; Table 1). Although only 6% of the bacterial and 3% of the archaeal FL-ASVs lacked genus-level homologs (≥94.5% identity) in SILVA, 38% of the bacterial and 14% of the archaeal FL-ASVs were without references at the species level (≥98.7% identity) indicating important novelty within AsCoM at this taxonomic level.

**Table 1:** Novel sequences and *de novo* taxa observed in the AsCoM reference database.

	**Sequence novelty** **(bacteria/archaea)**		**Taxonomy novelty** ^*^ **(bacteria/archaea)**	
	**Sequences**	**Percentage (%)**	***De novo* Taxa**	**Percentage (%)**
New phylum (<75.0%)	10/0	0.06/0	3/0	8.33/0
New class (<78.5%)	16/0	0.09/0	15/0	15.15/0
New order (<82.0%)	26/0	0.15/0	91/1	30.33/12.50
New family (<86.5%)	116/0	0.65/0	420/8	61.49/80.00
New genus (<94.5%)	1,138/4	6.36/3.03	1,997/16	80.95/84.21
New species (< 98.7%)	6,719/18	37.55/13.64	7,604/41	96.64/100

* *De novo* species also include known species that cannot be resolved based on full-length 16S rRNA genes.

Sequence novelty was determined based on the percent identity between each FL-ASV and their closest relative in the SILVA 138.1 SSURef NR99 database and taxonomic thresholds proposed by Yarza et al. (2014). Taxonomic novelty was defined based on the number of *de novo* taxa assigned by AutoTax at each taxonomic rank.

We then investigated the taxonomic novelty within the AsCoM database based on the percentage of AutoTax-assigned placeholder names (AsCoM_x_y) at the different taxonomic ranks (Figure 1; Table 1). For archaea, most taxa were assigned *de novo* placeholder names at the genus- and species level revealing a significant lack of taxonomic information in the lower ranks for homologs within the SILVA database. For bacteria almost 97% of all species, 81% of genera, 61% of families, and 30% of orders were assigned *de novo* placeholder names, revealing a great diversity of undescribed taxa within this ecosystem (Figure 1). This was especially apparent within the phyla of Bacteroidota, Chloroflexi, Myxococcota, Patescibacteria, Planctomycetota, and Proteobacteria (Figure 1; Supplementary Figure S1). The assignment of placeholder taxonomies to these sequences is essential for permitting informative community profiling because these taxa would otherwise be excluded from taxonomy-resolved studies. The benefits of this are most apparent at the lower taxonomic ranks, which are of biological importance given that species-level assignment can be required to distinguish between, e.g., beneficial, and pathogenic microbes (Berendsen et al., 2015; Elshafie and Camele, 2021; Garrido-Sanz et al., 2021).

### AsCoM provides improved references for plant microbiome samples

**Figure 2:**
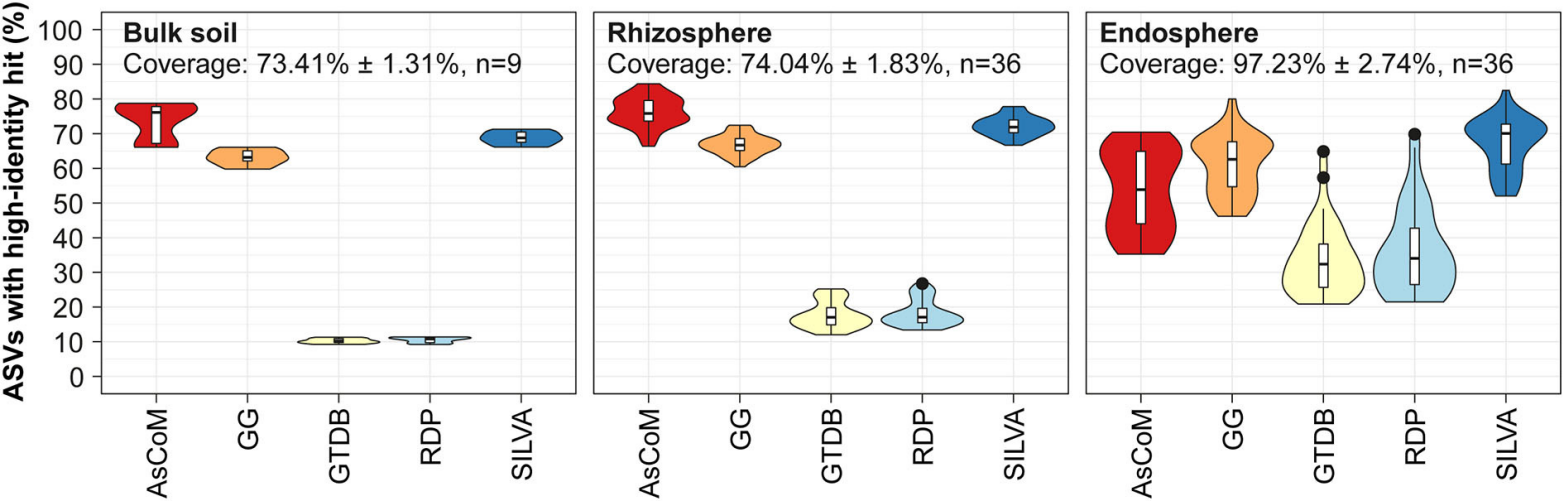
Database evaluation based on mapping of short-read amplicon data. V5–V7 ASVs obtained from Askov bulk soil as well as rhizosphere and endosphere samples from *Lotus japonicus, Medicago truncatula, Hordeum vulgare*, and *Zea mays* were mapped to the following reference databases and the proportion of high-identity hits (≥99% id) calculated: AsCoM, GreenGenes 16S 13.5 (GG) (Desantis et al., 2006), GTDB release 89 (Parks et al., 2020), RDP 16S v16 (Cole et al., 2014), and SILVA 138.1 SSURef NR99 (Quast et al., 2013). The ASVs were filtered based on their relative abundance (only ASVs with ≥0.01% relative abundance were kept) before the analyses. Coverage shows how much of the accumulated read abundance these abundant ASVs accounted for (mean ± standard deviation, number of samples).

Precise and comprehensive taxonomic classification requires a database with high-identity reference sequences for microbes within the ecosystem. To evaluate the coverage of the AsCoM database, we mapped 16S rRNA gene V5–V7 ASV data obtained from three different compartments (bulk soil, rhizosphere, and endosphere) recovered from *Hordeum vulgare, Zea mays, Medicago truncatula*, and *Lotus japonicus* grown in Askov soil to the AsCoM database as well as commonly applied universal reference databases and calculated the percentage of ASVs with high-identity (>99%) references in the databases (Figure 2). The V5–V7 region was used because popular primers targeting the V4 or V3–V4 region also amplify abundant plant chloroplast and mitochondria in the rhizo- and endosphere, dramatically reducing the number of bacterial reads and skewing their relative abundances (Beckers et al., 2016).

For bulk soil and rhizosphere samples, the AsCoM database (18,042 sequences) has more high-identity reference sequences (>99% identity) than the universal databases GreenGenes 16S v.13.5 (1,262,986 sequences) (Desantis et al., 2006), GTDB release 89 (145,904 sequences) (Parks et al., 2020), RDP 16S v16 (13,212 sequences) (Cole et al., 2014), and SILVA_138.1_SSURef_Nr99 (510,508 sequences) (Quast et al., 2013) (Figure 2). SILVA and GreenGenes were the best of the universal databases, likely reflecting a large number of reference sequences in those databases. For samples derived from the endosphere, the SILVA and GreenGenes databases contained a slightly higher proportion of high-identity reference sequences compared to AsCoM (Figure 2). This likely reflects a historical focus on the cultivation and isolation of bacteria from the endosphere, which has led to a high number of relevant reference sequences in the universal reference databases. Better coverage of these bacteria in AsCoM can be achieved by the incorporation of relevant sequences from SILVA. However, introducing sequences for SILVA, which may be of considerably lower quality than the chimera-free FL-ASVs created here, may reduce the overall reliability of the final database.

Because the AsCoM database was designed to cover both Askov soil and Cologne soil, we also evaluated the coverage of the database based on previously published amplicon data for Cologne soil (Thiergart et al., 2019). The overall picture was like that of the Askov dataset with the same percentage of high-identity references as in SILVA for bulk soil and rhizosphere samples and slightly lower coverage for the root microbiomes (Supplementary Figure S2).

### AsCoM improves taxonomic classification of ASVs from soil and plant compartments

**Figure 3:**
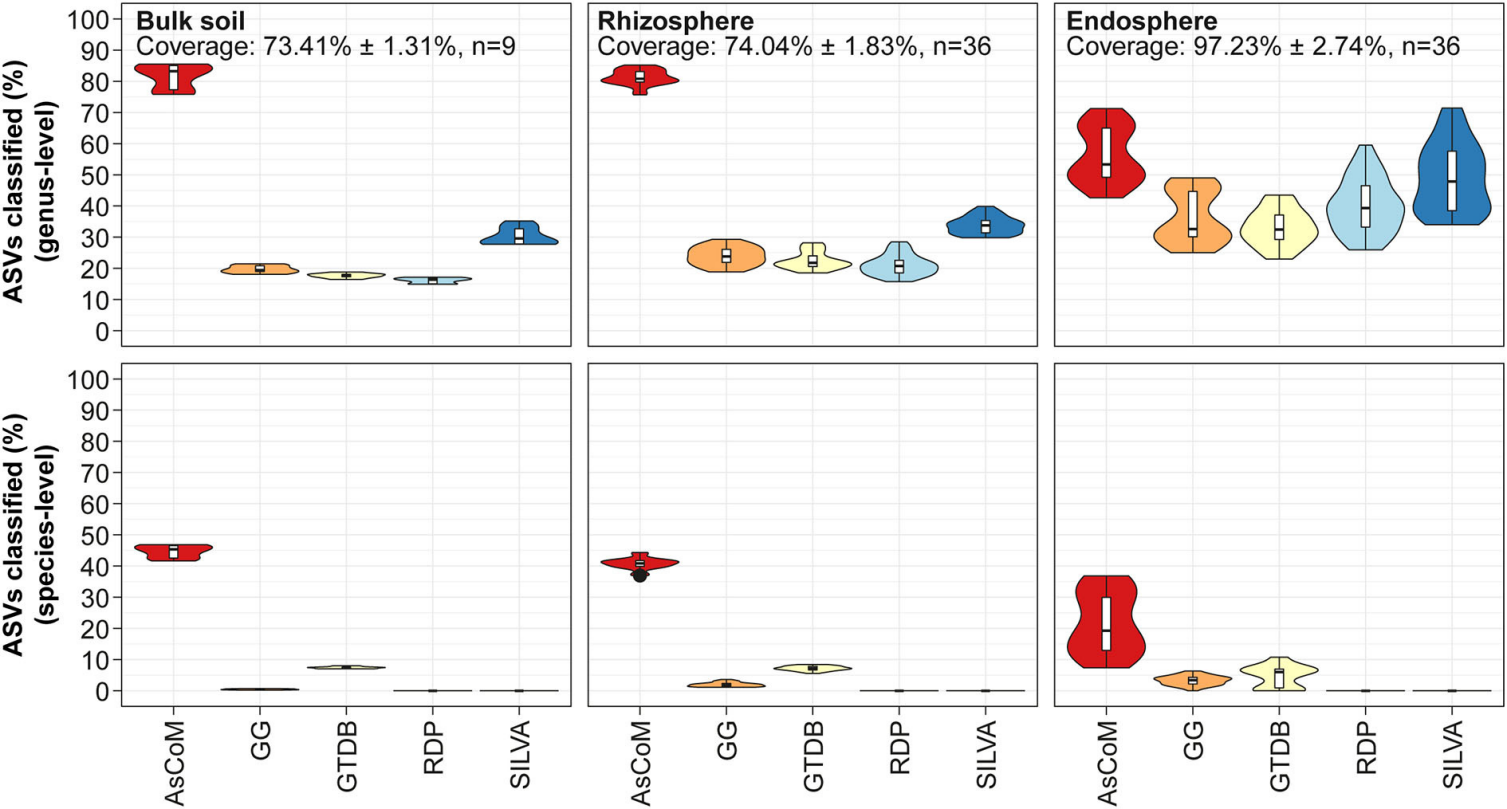
Database evaluation based on classification of short-read amplicon data. The V5–V7 amplicon data used in Figure 2 was classified using the SINTAX-classifier with AsCoM, GreenGenes 16S 13.5 (GG) (Desantis et al., 2006), GTDB release 89 (Parks et al., 2020), RDP 16S v16 (Cole et al., 2014), and SILVA 138.1 SSURef NR99 (Quast et al., 2013). The ASVs were filtered based on their relative abundance (only ASVs with ≥0.01% relative abundance were kept) before the analyses. Coverage shows how much of the accumulated read abundance these abundant ASVs accounted for (mean ± standard deviation, number of samples).

To evaluate the taxonomic classification performance of AsCoM, we classified the Askov V5–V7 ASVs using the SINTAX classifier and compared the classification at the genus- and species level with those of commonly used universal databases (Figure 3). The AsCoM database was superior at taxonomic classification both at genus- and species level (Figure 3).

A large proportion (up to 85%) of the ASVs from bulk soil and rhizosphere samples was classified at the genus level using AsCoM, representing a significant improvement over SILVA (up to 40%). The 15% of ASVs that were not assigned genus-level taxonomy by AsCoM can be explained by the relatively low phylogenetic signal contained within the V5–V7 amplicons (Dueholm et al., 2020). As we will see later, especially members of the families Comamonadaceae and Oxalobacteraceae were hard to differentiate at the genus level based on V5–V7 amplicons (Figure 5B).

**Figure 4:**
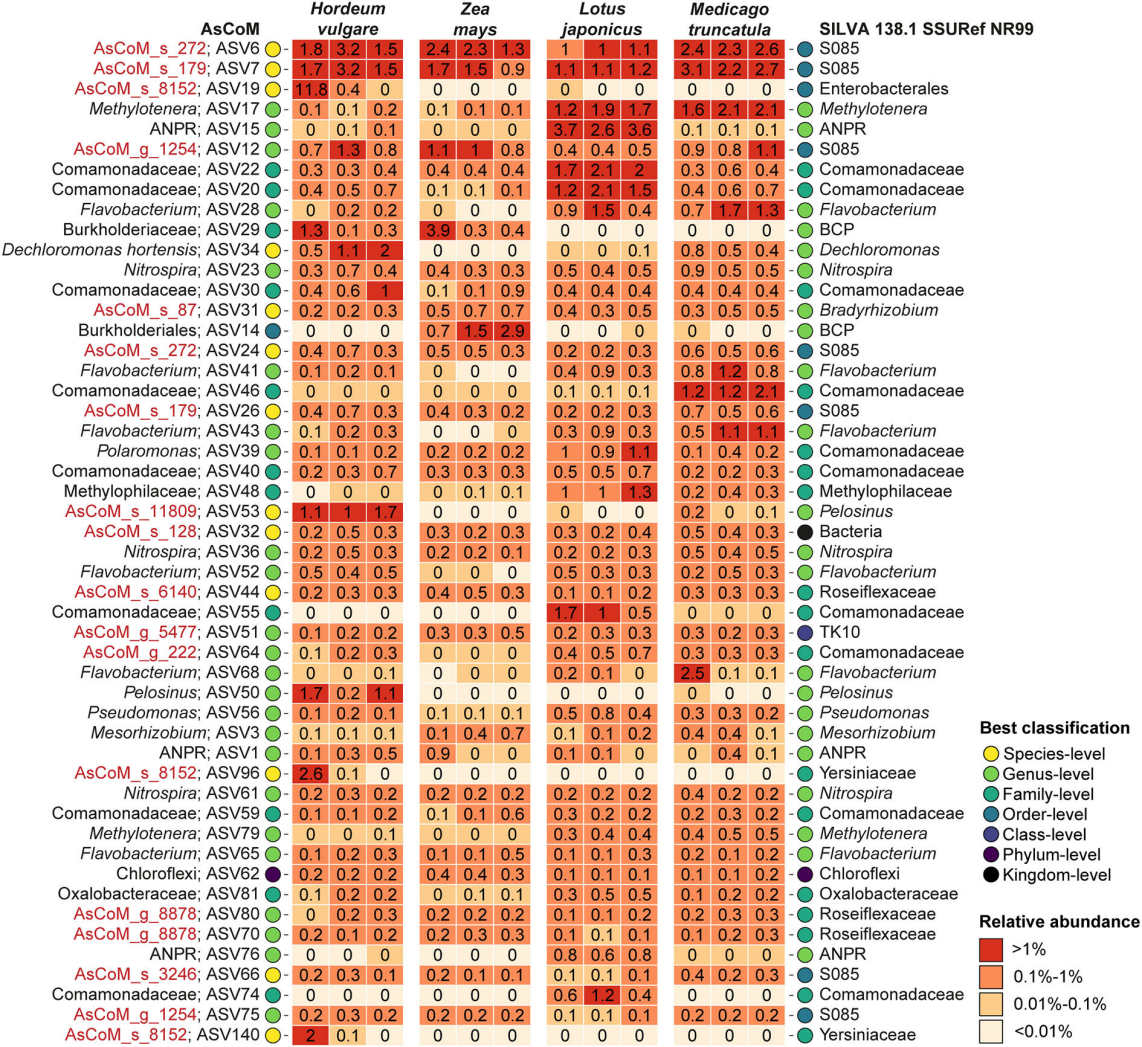
Classification of the 50 most abundant ASVs in the Askov rhizosphere with four plant types. ASVs were classified with the SINTAX-classifier using AsCoM and the SILVA 138.1 SSURef NR99 database and the best classification (lowest rank) is presented. Three biological replicates are shown for each plant type. *De novo* placeholder taxa provided by the AsCoM database is highlighted in red. ANPR, *Allorhizobium*-*Neorhizobium*-*Pararhizobium*-*Rhizobium*; BCP, *Burkholderia*-*Caballeronia*-*Paraburkholderia*.

Further analysis of the taxonomic assignments by AsCoM and SILVA demonstrated that the AsCoM database provided better classifications for the 50 most abundant rhizosphere ASVs across the four plant species, notably providing species-level classification for the three most abundant ASVs (Figure 4). This serves to highlight that the improved performance of AsCoM over universal databases includes abundant taxa that are likely to be of biological importance. Furthermore, these three abundant ASVs were assigned *de novo* placeholder species names by AutoTax, indicating that these ASVs are missing a comprehensive taxonomy in SILVA, and downstream analysis of these abundant isolates at lower taxonomic ranks would not be possible without the use of AsCoM.

Classification of the 50 most abundant ASVs in the endosphere across all four plant species was slightly improved at the genus level using the SILVA database compared to AsCoM (Supplementary Figure S3). However, AsCoM was able to provide species-level resolution for seven of these ASVs. This was not possible for any of the ASVs with SILVA. Most of the ASVs that were not classified at the genus level were from a few orders with many cultivated representatives, namely, Burkholderiales, Rhizobiales, and Enterobacteriales. The majority of the poorly classified ASVs represent isolates of lower abundance identified in the endosphere of barley and/or maize. This suggests that additional FL-ASVs recovered specifically from endosphere samples could improve the coverage of the AsCoM database in a later release.

Taxonomic classification of the Cologne amplicon dataset (Thiergart et al., 2019) with AsCoM and the universal reference databases showed clear improvements in the rate of genus- and species-level classification with AsCoM for bulk soil, rhizosphere, and root microbiome samples (Supplementary Figure S2).

### Taxonomic resolution and primer bias related to 16S rRNA V5–V7 amplicon sequencing

Despite the increasing interest and development of protocols for full-length 16S rRNA gene sequencing (Callahan et al., 2019; Jeong et al., 2021; Matsuo et al., 2021) and metagenomic methods (Milanese et al., 2019; Ye et al., 2019; Lu and Salzberg, 2020), short-read amplicon analysis remains popular for studying microbial communities due to the comparative low-sequencing costs and high accuracy. Drawbacks of the short-read approach are the lower phylogenetic signal provided by short reads and the amplification efficiency bias that primer pairs can exhibit toward certain taxa (Albertsen et al., 2015).

**Figure 5:**
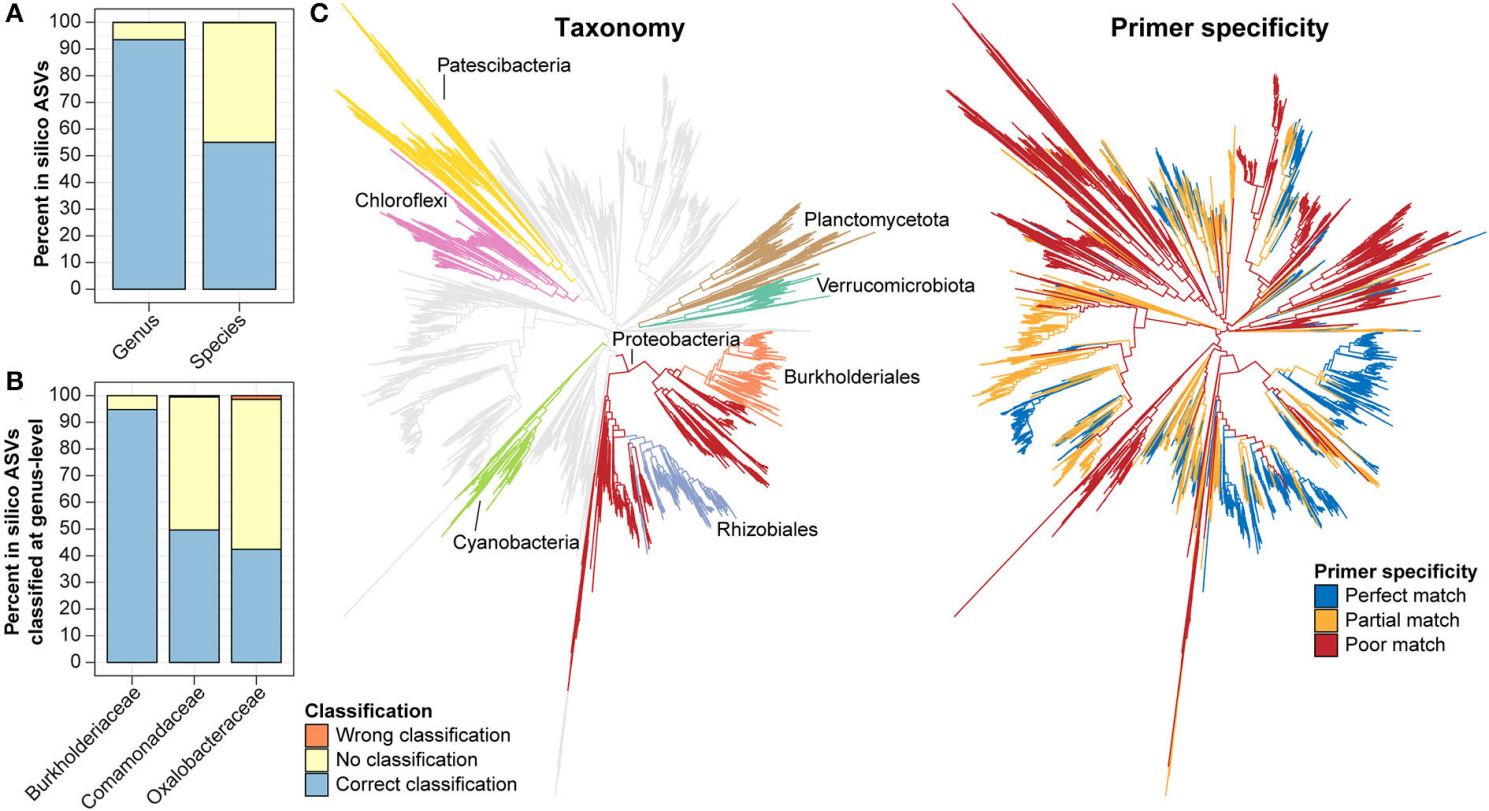
*In silico* evaluation of taxonomic resolution and primer mismatch of the V5–V7 primer. **(A,B)** *In silico* ASVs corresponding to the region amplified with the V5–V7 primer pair were extracted from all bacterial FL-ASVs **(A)** or selected families **(B)** in AsCoM and classified using the AsCoM database. Classifications were evaluated as correctly classified, wrongly classified, or not classified using the taxonomy of FL-ASV in the AsCoM database as the ground truth. **(C)** *In silico*, ecosystem-specific evaluation of primer specificity of the V5–V7 primer pair using Primer Prospector and bacterial FL-ASVs from the AsCoM database. The gray branches show perfect hits, the yellow branches show partial hits, and the red branches show poor hits (see Materials and Methods for definitions). The phyla most influenced by primer bias as well as the orders Burkholderiales and Rhizobiales are highlighted in different colors in the taxonomy tree.

Taking advantage of the AsCoM database, we evaluated the taxonomic resolution provided by V5–V7 ASVs, specifically regarding the microbial diversity found within Askov and Cologne soil. *In silico* ASVs corresponding to the V5–V7 region were extracted from the FL-ASVs in the AsCoM database. These ASVs were then classified using AsCoM using the SINTAX-classifier. Genus- and species-level classification of the *in silico* ASVs was compared to the taxonomy of their corresponding FL-ASVs, and the fraction of correctly or incorrectly classified ASVs was calculated (Figure 5A). We found that 93.5% of the *in silico* ASVs were correctly classified at the genus level, 6.5% were not classified, and only 0.07% was wrongly classified. Species-level classification was correctly provided for 55.1% of the ASVs, 44.8% could not be classified, and only 0.2% received wrong classifications. This confirms the high precision of classifications obtained using the AsCoM database.

In plant microbiome studies, the V5–V7 primer pair is a popular choice as plant-derived amplicons can be excluded from the sequencing pool. However, this feature was found to lead to lower coverage for bacteria compared to primers targeting the V3–V4 and the V4 region of the 16S rRNA gene when evaluated against the complete bacterial diversity in the SILVA database (Beckers et al., 2016). Because the AsCoM database only includes reference sequences from agricultural soil it can be used to evaluate the ecosystem-specific primer coverage and determine the bias toward amplification of certain bacterial groups resulting in underestimating the abundance or absence of certain bacterial groups within the bacterial community studied (Figure 5C). The *in-silico* primer evaluation showed that the V5–V7 primer pair is poorly suited for the study of many Cyanobacteria, Verrucomicrobiota, Planctomycetota, Chloroflexi, and Patescibacteria due to partial or poor primer matches. The primer pair does, however, provide perfect matches for most members of the Proteobacteria groups, especially the well-known plant-associated Burkholderiales and Rhizobiales (Figure 5C).

### Host preference of legumes and cereals

The AsCoM database allowed us to investigate signatures of host preference in the microbiota associated with the roots of the model legumes *Lotus japonicus* and *Medicago truncatula*, and the cereal crops *Hordeum vulgare* and *Zea mays*. To allow for a direct comparison between the host plants, all were grown concurrently in Askov unfertilized soil under controlled conditions.

Following three weeks of growth, rhizosphere and root endosphere compartments were harvested. For legumes, nodules were separated from the root to form an additional compartment for analysis. 16S rRNA gene amplicon sequencing was performed using the V5–V7 primers to minimize host organelle amplicon contamination, and the taxonomic assignment was performed using the AsCoM database. Alpha-diversity analysis showed reduced bacterial diversity within the endosphere compared to rhizosphere with a more severe reduction in diversity observed for legume plants compared to cereals (Supplementary Figure S4a). A larger variance was observed for the microbial diversity (inverse Simpsons) than for the richness (unique ASVs) between biological replicates for all soil samples. This reflects a heterogeneous microbial composition within the soil.

Beta-diversity analysis of the complete dataset revealed the separation of the samples primarily due to the compartments from which they were harvested with exception of legume endosphere and nodule samples which showed some overlap (Supplementary Figure S4b). Separation based on plant species was evident for the endosphere and nodule compartments, whereas all rhizosphere samples clustered closely with the bulk soil.

**Figure 6:**
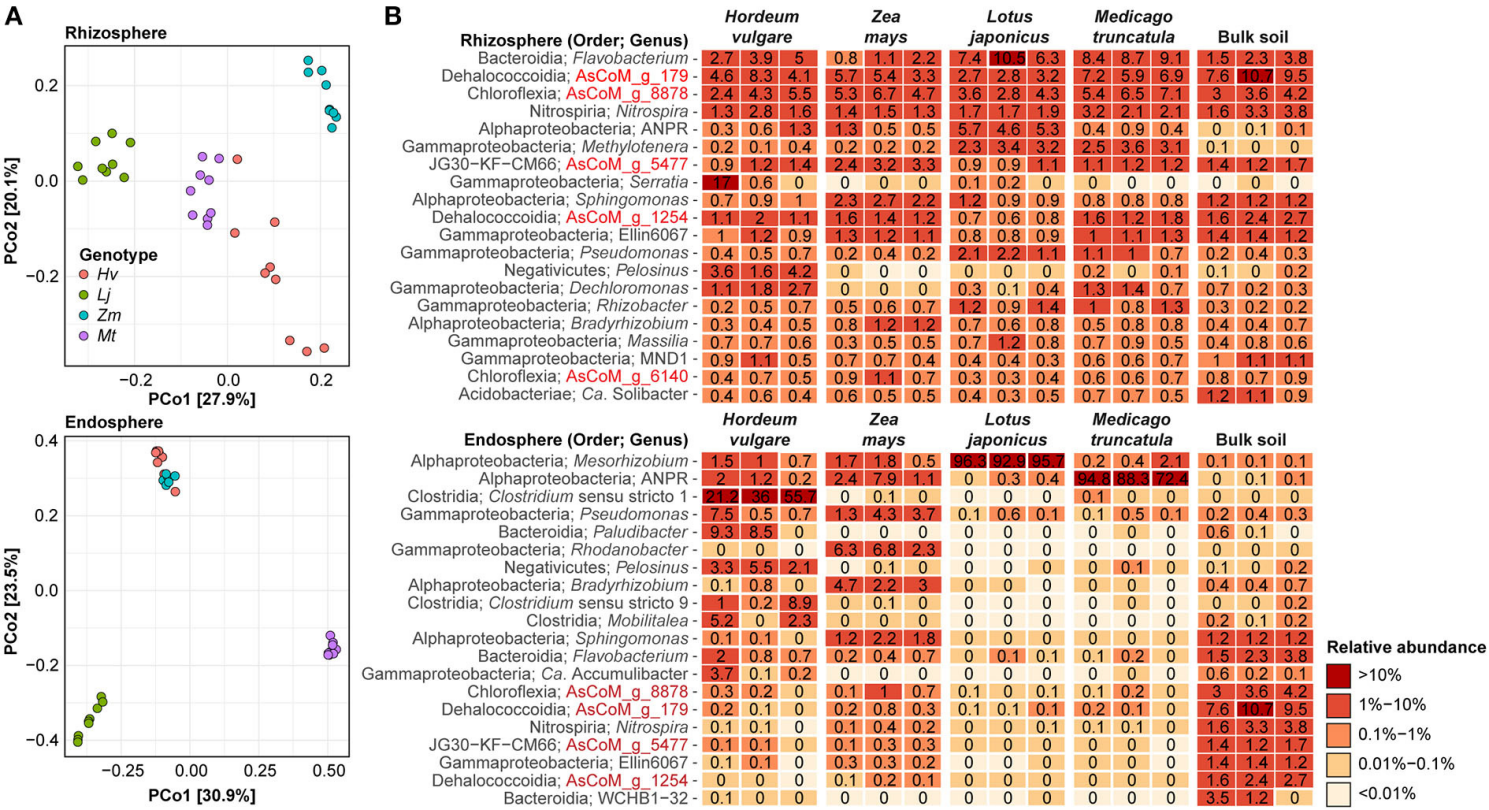
Effect of plant species on the rhizosphere and endosphere microbiota. **(A)** Beta-diversity analysis using Bray–Curtis dissimilarities separates samples on the basis of host species for both the rhizosphere and endosphere compartments. **(B)** Percent relative abundance of the 20 most abundant genera within the rhizosphere and endosphere compartments. Three biological replicates are shown for each host species. The relative abundance for bulk soil is included for reference.

To further investigate the specific effect of the host plants on rhizosphere and endosphere communities these compartments were further investigated separately. Both rhizosphere and endosphere communities show distinct clustering based on the host plant, except for the cereal endosphere samples which clustered together (Figure 6A). To gain additional information about the plant-specific microbiome, we took advantage of the improved taxonomic resolution afforded by the AsCoM database and investigated the microbial community structure of the different compartments at the genus level (Figure 6B). Host preference for specific rhizosphere microbes was evident, such as *Serratia* and *Pelosinus* for *H. vulgare, Sphingomonas* for *Z. mays*, and *Allorhizobium*-*Neorhizobium*-*Pararhizobium*-*Rhizobium* for *L. japonica*. Microbes with a preference for legumes over cereals were also identified, e.g., *Methylotenera*. Abundant rhizosphere genera with weak or no host preference according to comparable abundance across all hosts and bulk soil, included AsCoM_g_8878, AsCoM_g_179, *Flavobacterium*, and *Nitrospira*. Within the endosphere, host preferences were stronger than observed in the rhizosphere. The legume endospheres were dominated by symbiotic microbes, *Mesorhizobium* for *L. japonicus*, and *Allorhizobium*-*Neorhizobium*-*Pararhizobium*-*Rhizobium* for *M. truncatula* (Figure 6B). The cereals displayed more diversity within their endospheres. *Clostridium* is preferentially abundant in *H. vulgare*, while *Allorhizobium*-*Neorhizobium*-*Pararhizobium*-*Rhizobium, Pseudomonas, Rhodanobacter, Bradyrhizobium*, and *Sphingomonas* are most abundant in *Z. mays*.

The clear enrichment of compatible symbiotic rhizobia within legume endospheres is consistent with previous studies (Zgadzaj et al., 2016; Brown et al., 2020). To gain insight into the rhizobial colonization of cereals compared to legumes under the same experimental conditions, we investigated taxa annotated as belonging to the order Rhizobiales. Although the relative abundance of rhizobial taxa within cereals is greatly reduced compared to legumes their presence is detected, including the symbionts identified from within the legume endospheres and nodules (Supplementary Figure S5).

Different species or strains within the same genus can have different host preferences (Ofek et al., 2014; Tovi et al., 2019). Therefore, we also investigated host preference at the ASV level, which represents the highest phylogenetic resolution afforded by the amplicon data. We identified specific ASVs that were significantly enriched or reduced in the rhizosphere of the four plant species compared to bulk soil (Supplementary Figure S6; Supplementary Data S2). We found that for all plants, more ASVs were enriched than reduced in the rhizosphere compared to the bulk soil, and a higher number of ASVs were enriched for the legumes than for the cereal crops (1,275 vs. 366, respectively) (Supplementary Figure S6). This suggests that more bacteria are recruited by the legumes compared to the cereals. A large proportion of the enriched ASVs were classified at the species (27–35%) and genus level (67–80%) with AsCoM, again highlighting the unique taxonomic resolution afforded by this database. The enriched ASVs were not taxonomically restricted but covered more than 100 different families. However, the majority of the ASVs were affiliated with families that include known rhizobacteria, including Comamonadaceae (219 enriched ASVs across all plant species), Oxalobacteraceae (160 ASVs), Roseiflexaceae (119 ASVs), Flavobacteriaceae (95 ASVs), and Rhizobiaceae (80 ASVs) (Data S2). Interestingly, at the genus level, we found that most enriched ASVs were classified as the *de novo* genus AsCoM_g_8878 (113 ASVs) belonging to the family Roseiflexaceae. ASVs from this genus were enriched in the rhizosphere of all plant species except *H. vulgare* and should, therefore, be a target for further investigations. Other well-represented genera included *Flavobacterium* (95 ASVs), *Allorhizobium*-*Neorhizobium*-*Pararhizobium*-*Rhizobium* (48 ASVs), *Rhizobacter* (37 ASVs), and *Devosia* (36 ASVs).

## Discussion

The development and use of ecosystems-specified 16S rRNA reference databases, such as AsCoM, enables increased taxonomic resolution in amplicon sequencing studies and provides a taxonomic framework for comparing data obtained using primer pairs targeting different regions of the 16S rRNA gene. A major contribution to the improved classification of ASVs at the genus- and species level is the *de novo* placeholder taxonomy created in the reference database with AutoTax for environmental lineages that do not have any official taxonomy at lower ranks (Dueholm et al., 2020). Because the *de novo* taxonomy is assigned based on fixed identity thresholds for each taxonomic rank, it does not take different rates of evolution across the tree of life into account. It should, therefore, not be considered a replacement for proper phylogenetic classification, which ideally requires phylogenomic analyses (Hug et al., 2016; Parks et al., 2018, 2020; Zhu et al., 2019). However, the placeholder taxonomy allows us to identify new ecological relevant lineages that should be targeted for the recovery of high-quality metagenome-assembled genomes (MAGs), phylogenomics, and further investigations (Nierychlo et al., 2021; Petriglieri et al., 2022; Singleton et al., 2022). A good example is the *de novo* genus AsCoM_g_8878 within the family Roseiflexaceae for which many ASVs displayed strong host enrichment. By submitting these MAGs to GTDB and making the FL-ASV publicly available for incorporation into SILVA, we can expand and improve the universal reference databases, providing a future benefit for the entire field.

A unique feature of the ecosystem-specific databases created with AutoTax is that they are essentially chimera- and error-free. The attachment of unique molecular identifiers (UMIs) to each end of the original 16S rRNA gene template molecule before any PCR amplification steps allows the filtering of true biological sequences from chimera already in the synthetic long-read assembly (Karst et al., 2018; Dueholm et al., 2020). The few sequencing errors that may occur after this initial quality control are all low abundant, and these are removed when FL-ASVs are subsequently resolved, as previously shown using mock communities (Dueholm et al., 2020). The extreme quality of the reference database makes it ideal for lineage-specific probe design. The development of species-specific fluorescent *in situ* hybridization (FISH) probes provides opportunities to visualize the morphology and spatial arrangement of individual species in complex samples and can be combined with Raman microspectroscopy to elucidate their activity and metabolic traits *in situ* (Wagner et al., 2006; Huang et al., 2007; Singer et al., 2017; Fernando et al., 2019).

Another important aspect of the AsCoM database is that it allows us to determine the ecosystem-specific taxonomic resolution afforded by different 16S rRNA gene-based amplicons, and the expected primer bias introduced by the primers used for amplification. This insight is important for making sound conclusions, and it may also form the basis for the future development of improved amplicon strategies.

## Data availability statement

All sequencing data have been submitted to the Sequence Read Archive under the project ID PRJNA787301. Details about individual datasets can be found in Supplementary Data S1. Data were analyzed with R v.4.0.5 (R Development Core Team, 2008) using RStudio IDE (RStudio Team, 2020), with the tidyverse v.1.3.1 (Wickham et al., 2019), vegan v.2.5.7 (Oksanen et al., 2015), Ampvis2 v.2.7.9 (Andersen et al., 2018), patchwork v. 1.1.1. (Pedersen, 2020), and ggtree v. 3.1.1.991 (Yu et al., 2017) packages. All R scripts used for data analysis and visualization, the AsCoM reference database in SINTAX and QIIME format, and an ARB-database with the raw alignment, filters, and phylogenetic trees are available at GitHub: https://github.com/msdueholm/Publications/tree/master/Overgaard2022a.

## Author contributions

KT, SZ, BC, and ZB provided samples. CO prepared sequencing libraries for full-length 16S rRNA sequencing. CO and MD processed the full-length 16S rRNA sequences. KT, SZ, ZB, and SK performed V5-7 amplicon sequencing. CO, MD, and SK performed the bioinformatic analyses. CO, MD, SR, and SK wrote the manuscript and designed the study. MD, SR, and SK supervised the study. All authors read and approved the final manuscript.

## Funding

This study was funded by Independent Research Fund Denmark (Grant no. 9041-00236B).

## Conflict of interest

The authors declare that the research was conducted in the absence of any commercial or financial relationships that could be construed as a potential conflict of interest.

## Publisher's note

All claims expressed in this article are solely those of the authors and do not necessarily represent those of their affiliated organizations, or those of the publisher, the editors and the reviewers. Any product that may be evaluated in this article, or claim that may be made by its manufacturer, is not guaranteed or endorsed by the publisher.
