# Supplementary material for: Application of ecosystem-specific reference databases for increased taxonomic resolution in soil microbial profiling
Source: Front Microbiol. 2022 Nov 3;13:942396. doi: 10.3389/fmicb.2022.942396 (PMC9669317; doi:10.3389/fmicb.2022.942396)
Supplement: Supplementary file 1 [file Data_Sheet_1.PDF]

Supplementary Material

1 Supplementary Figures

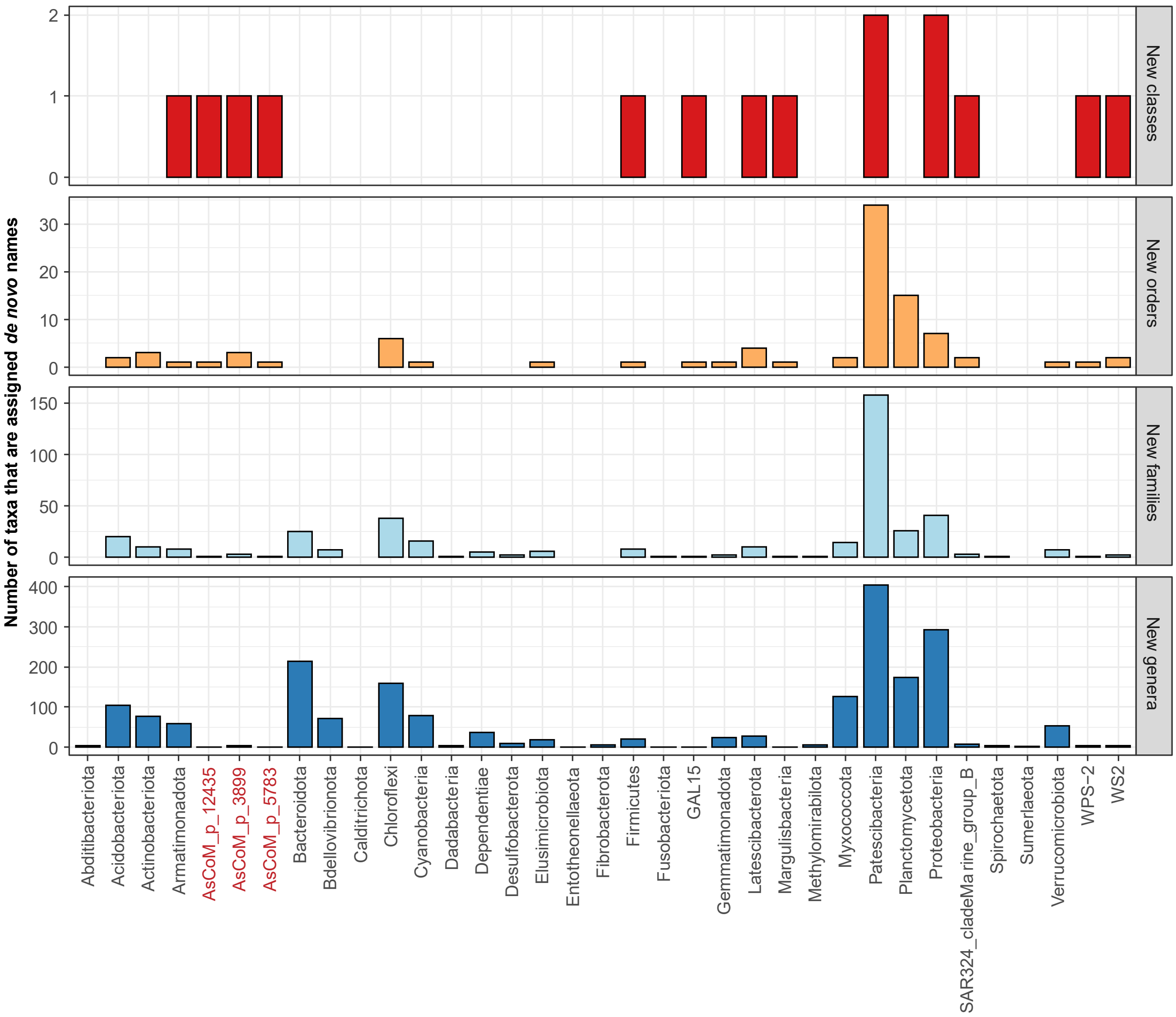

Supplementary Figure 1. Taxonomic novelty within different phyla in the AsCoM database. The number of taxa assigned de novo name by AutoTax within the taxonomic rank of class, order, family, and genus for each phylum. De novo phyla are highlighted in red.

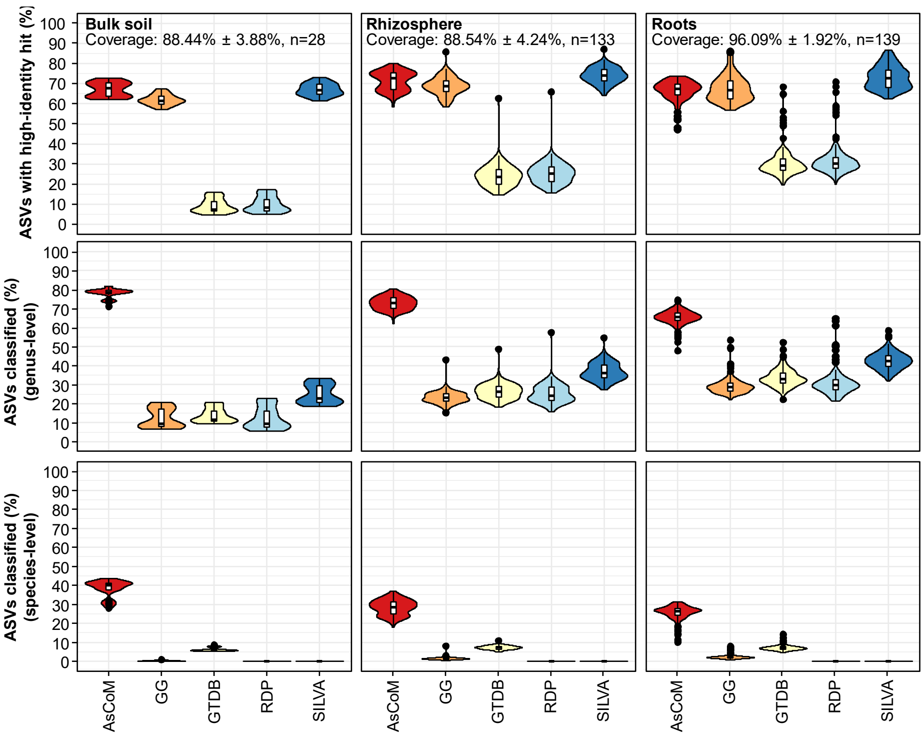

Supplementary Figure 2. Database evaluation based on mapping of short-read amplicon data from Cologne soil. Raw V5-V7 amplicon data was obtained from Thiergart et al., (2019) and processed as described for the Askov data in the main article. The data was separated based on the sample compartment (Bulk soil, rhizosphere, and root microbiota), and mapped to or classified with the following reference databases to determine the percentage of high-identity hits (≥99% id) and genus- and species-level classifications: AsCoM, GreenGenes 16S 13.5 (GG) (Desantis et al., 2006), GTDB release 89 (Parks et al., 2020), RDP 16S v16 (Cole et al., 2014), and SILVA 138.1 SSURef NR99 (Quast et al., 2013). The ASVs were filtered based on their relative abundance (only ASVs with ≥0.01% relative abundance in individual samples were kept) before the analyses. Coverage shows how much of the accumulated read abundance these abundant ASVs accounted for (mean ± standard deviation, number of samples).

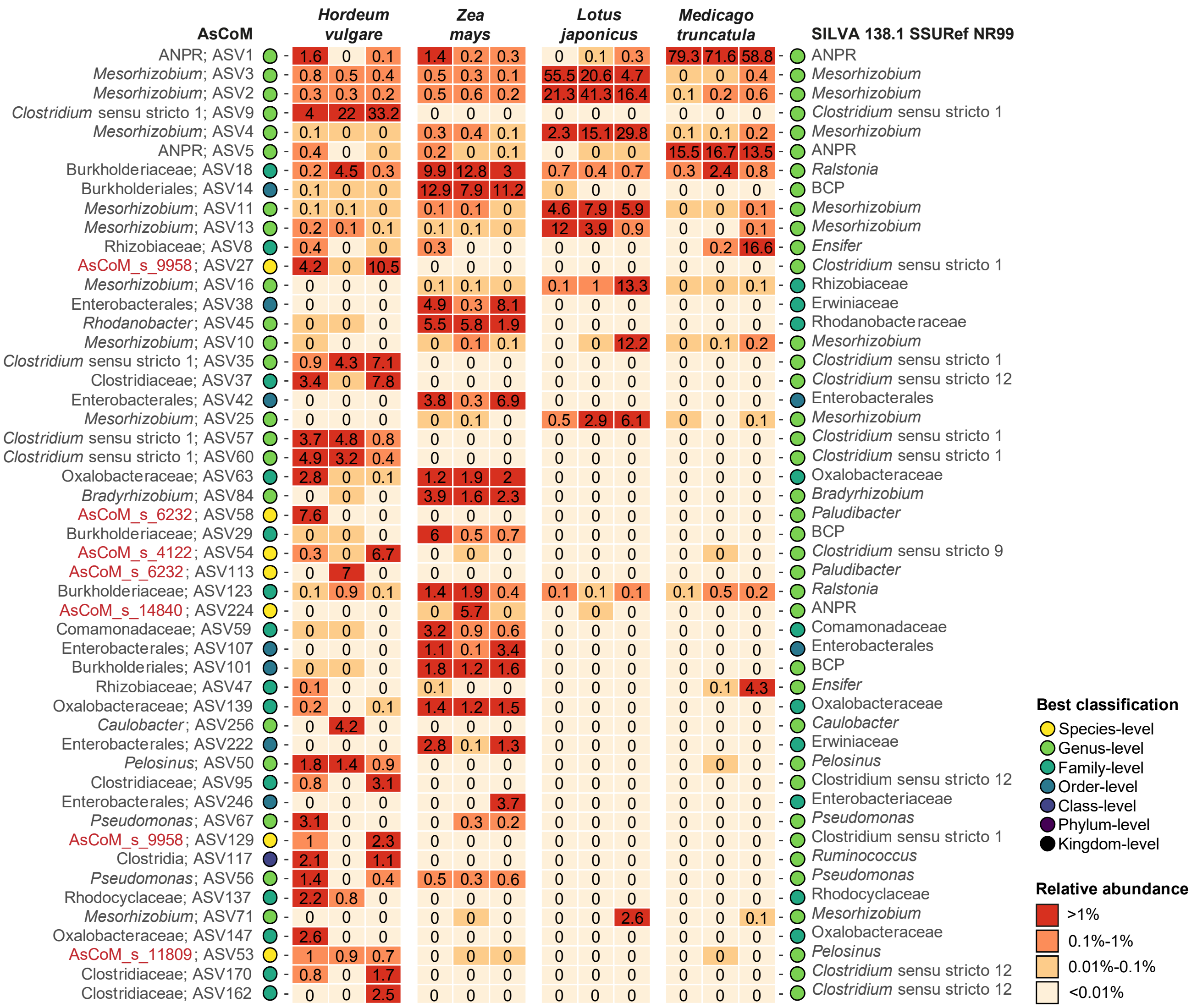

Supplementary Figure 3. Classification of the 50 most abundant ASVs in the Askov endosphere with four plant types. ASVs were classified with the SINTAX-classifier using AsCoM and the SILVA 138.1 SSURef NR99 database and best classification (lowest rank) is presented. Three biological replicates are shown for each plant type. De novo placeholder taxa provided by the AsCoM database is highlighted in red. ANPR: *Allorhizobium-Neorhizobium-Pararhizobium-Rhizobium*; BCP: *Burkholderia-Caballeronia-Paraburkholderia*.

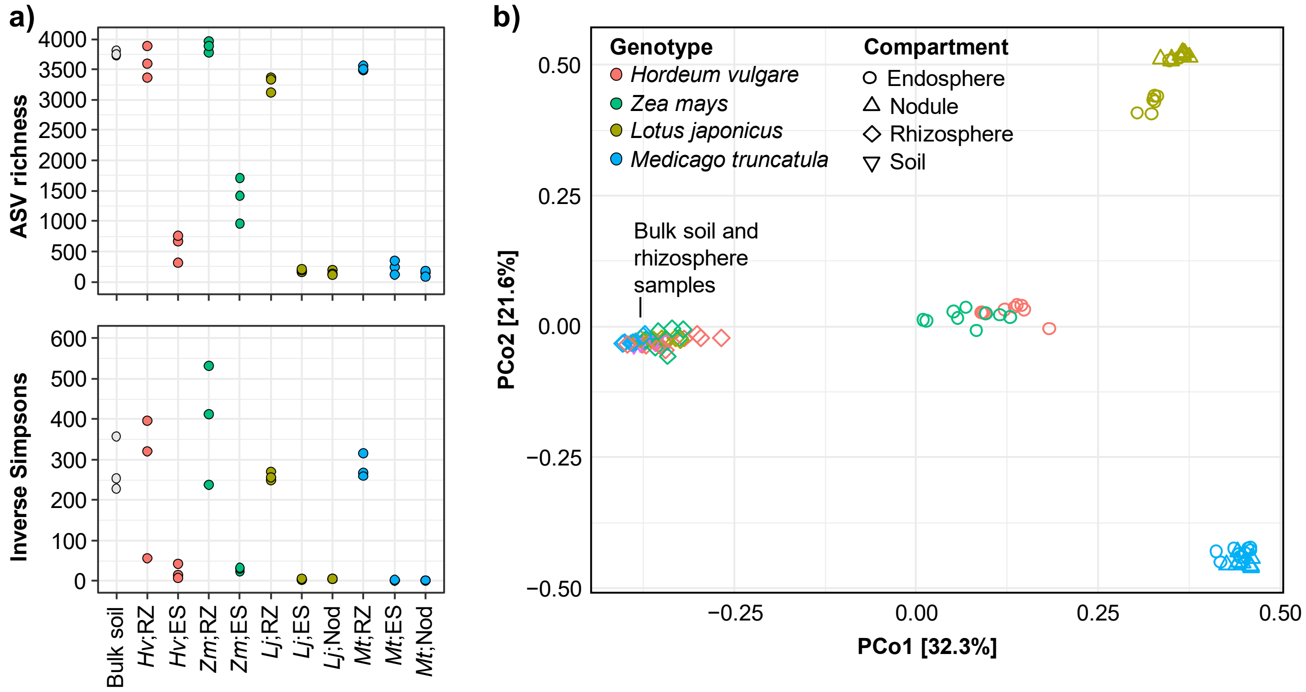

Supplementary Figure 4. Diversity metrics. a) Alpha-diversity analysis using the ASV richness and the inverse Simpson's index of bulk soil, rhizosphere (RZ), endosphere (ES) and nodule (Nod) compartments harvested from *Lotus japonicus* (*Lj*), *Medicago truncatula* (*Mt*), *Hordeum vulgare* (*Hv*) and *Zea mays* (*Zm*). Each dot represents the average of three technical replicates. b) Beta-diversity analysis using Bray-Curtis dissimilarities of the same microbiome samples.

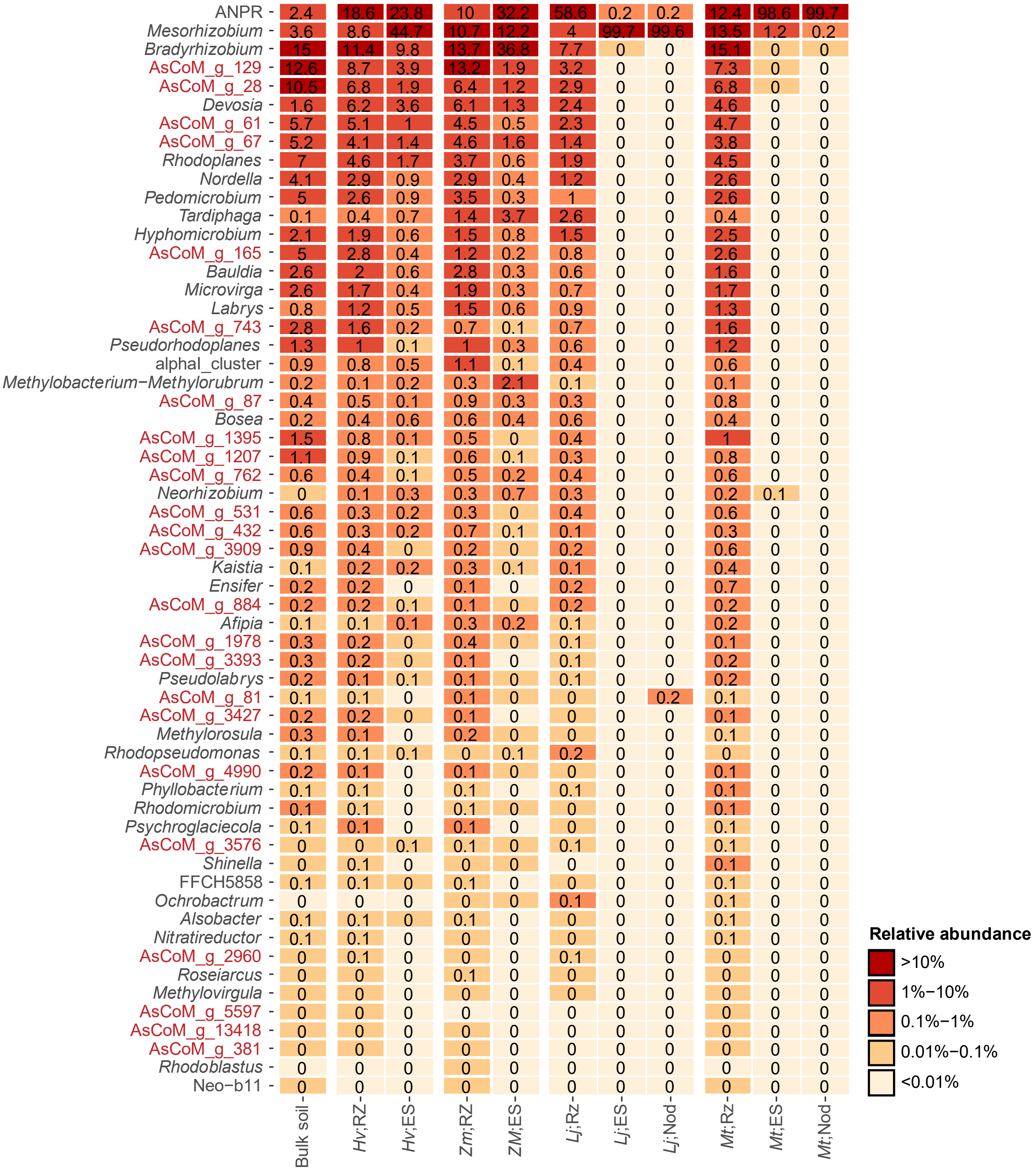

Supplementary Figure 5. Host preference for rhizobia. Relative abundance of all genera in the Order Rhizobiales across the rhizosphere (RZ), endosphere (ES) and nodule (Nod) compartments harvested from *Lotus japonicus* (*Lj*), *Medicago truncatula* (*Mt*), *Hordeum vulgare* (*Hv*) and *Zea mays* (*Zm*). Bulk soil is included for reference. The means of three biological replicates are shown. De novo placeholder taxa provided by the AsCoM database are highlighted in red. ANPR: *Allorhizobium-Neorhizobium-Pararhizobium-Rhizobium*.

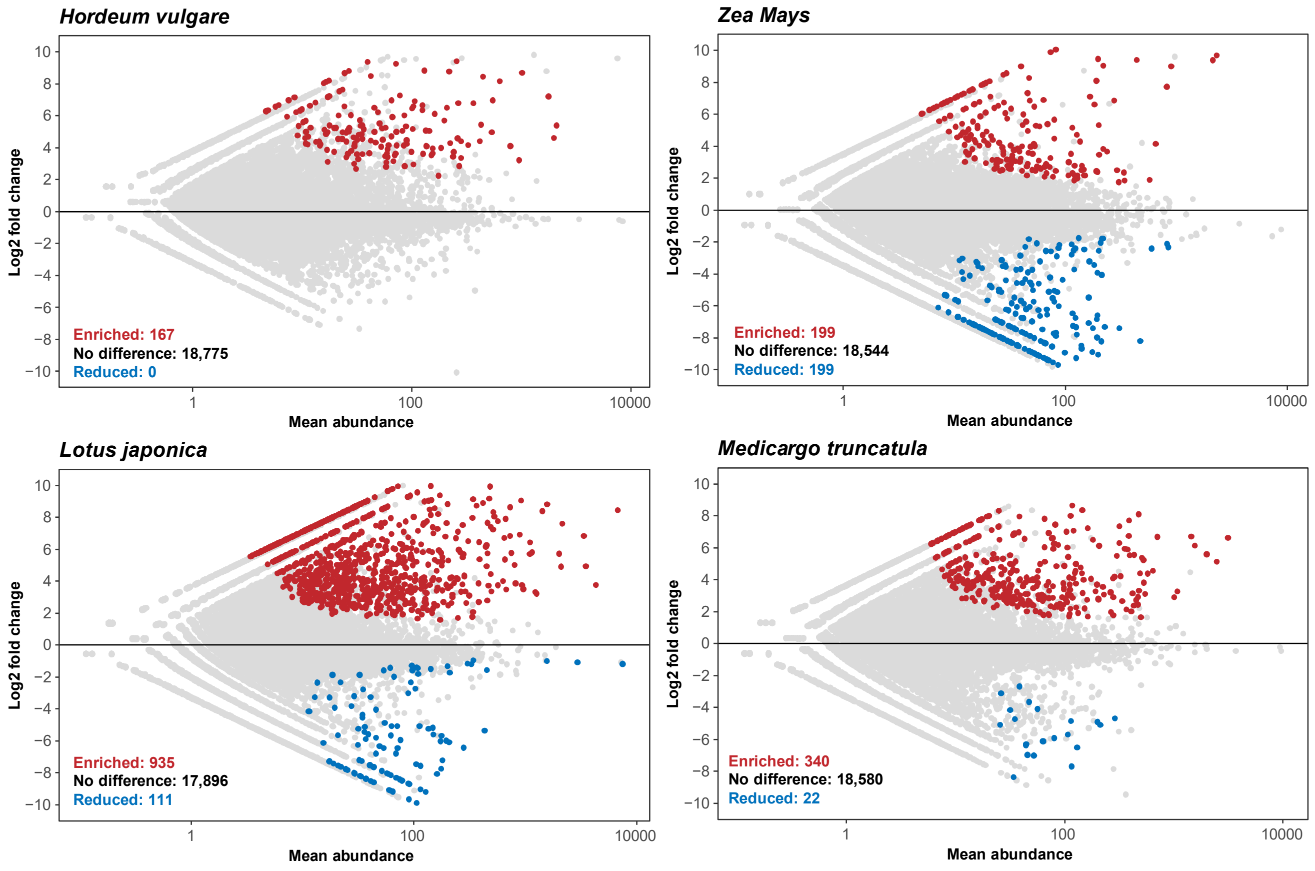

Supplementary Figure 6. MA-plot of ASVs enriched in the rhizosphere of four plant species compared to bulk soil. DEseq2 analysis was used to determine which ASVs were enriched in the rhizosphere. ASVs having a significant (padj < 0.01) positive log2 fold change are enriched in the rhizosphere compared to bulk soil (red) whereas ASVs having a significant (padj < 0.01) negative log2 fold change are reduced (blue). The adjusted p-values, log2 fold change, and taxonomy for individual ASVs can be found in Data S2.

2 Supplementary Tables

Supplementary Table 1: Oligonucleotides used for full-length 16S rRNA gene library preparation. Unique molecular tags, and sample barcodes are marked with blue and red, respectively. Primers specific for bacteria or archaea are marked with "_b_" or "_a_" in their names, respectively.

Name	Sequence (5' to 3')
f16S_b_pcr1_fw1	CTCCACCCAGACTCATCCATNNNNNNNNNNNNNNNTGCCTCTTAGAGTTTGATCMTGGCTCAG
f16S_b_pcr1_fw2	CTCCACCCAGACTCATCCATNNNNNNNNNNNNNNNTCCTCTACAGAGTTTGATCMTGGCTCAG
f16S_b_pcr1_fw3	CTCCACCCAGACTCATCCATNNNNNNNNNNNNNNNTCATGAGCAGAGTTTGATCMTGGCTCAG
f16S_b_pcr1_fw4	CTCCACCCAGACTCATCCATNNNNNNNNNNNNNNNCCTGAGATAGAGTTTGATCMTGGCTCAG
f16S_b_pcr1_rv1	AGCGCGGCAAAGATGAAGATNNNNNNNNNNNNNNNTGAACCTTGACGGGCGGTGWGTRCA
f16S_a_pcr1_fw1	CTCCACCCAGACTCATCCATNNNNNNNNNNNNNNNTGCCTCTTTCCGGTTGATCCYGCBRG
f16S_a_pcr1_fw2	CTCCACCCAGACTCATCCATNNNNNNNNNNNNNNNTCCTCTACTCCGGTTGATCCYGCBRG
f16S_a_pcr1_fw3	CTCCACCCAGACTCATCCATNNNNNNNNNNNNNNNTCATGAGCTCCGGTTGATCCYGCBRG
f16S_a_pcr1_fw4	CTCCACCCAGACTCATCCATNNNNNNNNNNNNNNNCCTGAGATTCCGGTTGATCCYGCBRG
f16S_a_pcr1_rv1	AGCGCGGCAAAGATGAAGATNNNNNNNNNNNNNNNTGAACCTTGGCCATGCAMYWCCTCTC
f16S_pcr2_fw	CTCCACCCAGACTCATCCAT
f16S_pcr2_rv	AGCGCGGCAAAGATGAAGAT
f16S_readtag_fw	CAAGCAGAAGACGGCATACGAGATGTGACTGGAGTTCAGACGTGTGCTCTTCCGATCTCTCCACCCAGACTCATCCAT
f16S_readtag_rv	CAAGCAGAAGACGGCATACGAGATGTGACTGGAGTTCAGACGTGTGCTCTTCCGATCTAGCGCGGCAAAGATGAAGAT
f16S_b_linktag_fw	CAAGCAGAAGACGGCATACGAGATCGGTCTCGGCATTCCTGCTGAACCGCTCTTCCGATCTCTGAGCCAKGATCAAACTCT
f16S_b_linktag_rv	AATGATACGGCGACCACCGAGATCTACACTCTTTCCCTACACGACGCTCTTCCGATCTTGYACWCACCGCCCGTC
f16S_a_linktag_fw	CAAGCAGAAGACGGCATACGAGATCGGTCTCGGCATTCCTGCTGAACCGCTCTTCCGATCTCYVGCRGGATCAACCGGA
f16S_a_linktag_rv	AATGATACGGCGACCACCGAGATCTACACTCTTTCCCTACACGACGCTCTTCCGATCTGAGAGGWRKTGCATGGCC
f16S_read2_fw	GCTCTTCCGATCTCTCCACCCAGACTCATCCAT
f16S_read2_rv	GCTCTTCCGATCTAGCGCGGCAAAGATGAAGAT
